# Confined Crystallization of Thin Plasma-Polymerized Nanocomposite Films with Maleic Anhydride and Cellulose Nanocrystals under Hydrolysis

**DOI:** 10.3390/molecules27175683

**Published:** 2022-09-03

**Authors:** Pieter Samyn

**Affiliations:** 1 Bio-Based Materials Engineering, University of Freiburg, Werthmannstrasse 6, 79085 Freiburg, Germany; pieter.samyn@outlook.be; 2 SIRRIS Smart Coatings Application Lab, Department of Circular Economy and Renewable Materials, Wetenschapspark 3, 3590 Diepenbeek, Belgium

**Keywords:** plasma, polymerization, nanocellulose, maleic anhydride, crystallization

## Abstract

The creation of novel surface morphologies through thin-film patterning is important from a scientific and technological viewpoint in order to control specific surface properties. The pulsed-plasma polymerization of thin nanocomposite films, including maleic anhydride (MA) and cellulose nanocrystals (CNC), may result in different metastable film morphologies that are difficult to control. Alternatively, the transformation of deposited plasma films into crystalline structures introduces unique and more stable morphologies. In this study, the structural rearrangements of plasma-polymerized (MA+CNC) nanocomposite films after controlled hydrolysis in a humid atmosphere were studied, including effects of plasma conditions (low duty cycle, variable power) and monomer composition (ratio MA/CNC) on hydrolysis stability. The progressive growth of crystalline structures with fractal dendrites was observed in confined thin films of 30 to 50 nm. The structures particularly formed on hydrophilic substrates and were not observed before on the more hydrophobic substrates, as they exist as a result of water penetration and interactions at the film/substrate interface. Furthermore, the nucleating effect and local pinning of the crystallites to the substrate near CNC positions enhanced the film stability. The chemical structures after hydrolysis were further examined through XPS, indicating esterification between the MA carboxylic acid groups and CNC surface. The hydrolysis kinetics were quantified from the conversion of anhydride groups into carboxylic moieties by FTIR analysis, indicating enhanced hydrolytic stability of p(MA+CNC) nanocomposite films relative to the pure p(MA) films.

## 1. Introduction

The deposition of thin polymer films enables the modification of chemical and functional surface characteristics, as nanoscale structuring can be employed for the creation of distinct patterns with tailorable properties for wetting [1], anti-fouling [2], optical appearance [3], adhesion [4], environmental sensitivity [5], bacterial/cell interactions [6] and antimicrobial or bioresponsive properties [7]. The basics for plasma polymerization of polymer thin films are known [8], but proper control of morphologies and stability remains challenging. In a top-down approach, plasma micropatterns were created for the immobilization of chemical functionalities [9]. In a bottom-up approach, the plasma conditions can be controlled to form polymer nanostructures [10]. Often, the lack of specific nucleation sites during plasma polymerization is a major problem in handling their morphologies and properties. In contrast, the controlled growth of structured nanocomposite films and multifunctional architectures from solution starts from nucleation sites that might be introduced under forms of nanocellulose particles [11]. 

Maleic anhydride (MA) is a multifunctional and biocompatible polymer that can be deposited by plasma polymerization [12]. In order to maximize the retention of functional groups in plasma-polymerized p(MA) films, a critical balance of plasma parameters should be selected with low nominal power or low pulsing times [13,14,15]. The film formation is governed by gas-phase dissociation and growth processes, which need to be narrowly controlled in parallel with complex interactions in the plasma reactor [16,17]. Under low duty cycles, however, the p(MA) film stability (e.g., mechanical, hydrolysis) is relatively low due to its low cross-linking density. The p(MA) film properties can be further tailored through variations of polymer chemistry, post-functionalization, or other post-treatment processes [18]. Alternatively, the encapsulation of nanofillers interferes with the plasma polymerization reactions and influences the film morphology [19]. Although the engineering of plasma films, including inorganic (e.g., silver) nanoparticles, was frequently performed [20], it has also been shown before that several morphologies of p(MA) films were obtained by adding cellulose nanocrystals (CNC) [21]. However, the formation of nanostructured films through buckling phenomena was unstable and difficult to obtain [22]. Therefore, the stability and transformation of such nanocomposite films should be further controlled.

The stability of plasma-polymerized films may be affected in presence of water [23], changing pH or ionic strength [24,25]. Recent efforts were made in reducing the aging phenomena in plasma polymer films [26], while controlled aging may alternatively result in the formation of unique surface structures. In general, the interaction of thin films with water leads to a relaxation of internal compressive stresses, yielding unstable film morphologies such as blisters, folds and wrinkles [27]. Owing to the hydrophilic properties of maleic anhydride, water sensitivity depends on the internal network structure of the polymerized film. In particular, the effects of aqueous solutions on plasma films deposited at low-duty-cycle conditions were investigated [28]; the mechanisms for water interaction with plasma films include swelling [29], removal of non-polymerized monomers and hydrolysis reactions [30]. The latter are enhanced through the easy diffusion of water through a weakly cross-linked polymer network and interaction with polar anhydride groups through hydrogen bonding between water and the polar groups [31]. The hydrolysis results in a progressive loss of the functional anhydride groups with time [32], but the resistance against hydrolysis was improved for films deposited at high power or long times corresponding to the ticker films [33]. With the change in relative humidity, water penetration and uptake in p(MA) films resulted in reversible swelling due to interactions between water and the carboxylic groups [34]. The detailed nanoscale characterization of water penetration through plasma-polymerized coatings inferred that the diffusion of water towards the interface with the substrate was crucial in the destabilization of hydrophilic films [35]. The controlled hydrolysis of p(MA) films allows for an increase in the reactivity of the polymer film and can be applied, e.g., on further attachment of silver nanoparticles [36] or controlled gelation for biocompatible hydrogels [37].

The processes of film reorganization under hydrolysis need an understanding of combined wetting dynamics and crystallization phenomena [38]. The solid-state dewetting of thin films resulted from instabilities leading to pattern formation and film stabilization [39]. The crystallization dewetting was observed in thin films (sub-100 nm range) of polymer blends [40] or block copolymers [41]. The crystallization phenomena are steered by nucleation, crystal formation and growth, while being controlled by the overall thermodynamics of the system [42]. However, competition between crystallization and dewetting in thin polymer films mostly occurs in specific temperature ranges [43]. Alternatively, molecular changes and enhanced molecular mobility in the polymer chain during hydrolysis also introduce structural rearrangements and eventual crystallization [44]. The morphology of polymers crystallized in confined systems is different from the bulk properties, as reviewed before [45,46,47]. Hence, the creation of novel crystallization morphologies under nanoscale confinement is of importance for tailoring polymer properties or creating new functionalities [48].

As the plasma deposition of nanocomposite films of p(MA+CNC) previously resulted in unstable film morphologies that were difficult to control [49], the hydrolysis stability and conversion into stable crystalline structures are further investigated in this work. The hydrolysis kinetics are analyzed as a function of monomer composition and plasma conditions (low duty cycle, various power), where the favorable effects of CNC fillers on hydrolysis stability are illustrated and unique crystalline morphologies were obtained. This study provides evidence for creating stable patterns after controlled post-treatment of plasma-deposited nanocomposite films, leading to a new and potentially useful way of surface structuring, e.g., for the future design of bio-interfaces.

## 2. Results

### 2.1. Morphological Analysis of Plasma-Deposited Coatings

**Figure 1:**
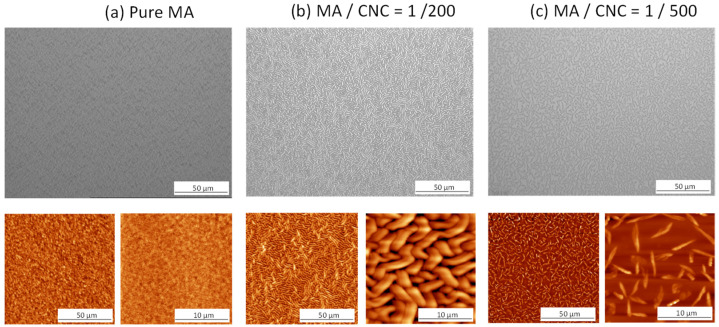
Morphology of pulsed-plasma-polymerized films with different composition, (**a**) pure maleic anhydride p(MA) film, (**b**) p(MA+CNC) nanocomposite film with MA/CNC = 1/200, (**c**) p(MA+CNC) nanocomposite film with MA/CNC = 1/500, according to optical microscopy (**top row**) and AFM (**bottom row**).

The morphologies of pulsed-plasma-polymerized maleic anhydride films (p(MA)) and plasma-polymerized nanocomposite film with CNCs (p(MA+CNC)) deposited onto the silicon substrates were recorded immediately after plasma deposition and are illustrated in Figure 1 through microscopic analysis and AFM images. Depending on the composition of the monomer feed, the morphology of the films and/or deposits can be highly altered.

The p(MA) film (Figure 1a) formed a homogeneous layer under present plasma conditions (P_p_ = 20 W, *DC* = 2%) with a thickness of the film of 30 ± 5 nm after given polymerization time. The present study only used a low *DC* value, as the effects of changing *DC* on the properties of a p(MA) film are already known [50]. Although the deposition was performed on a hydrophilized wafer surface in contrast with previous conditions deposited onto pure wafer surfaces [49], the observations were in agreement. The bare SiO_2_ wafers were characterized by a static water contact angle of 45°, while it decreased towards < 15° after activation treatment. The film thickness may depend on the exact positioning in the reactor, deposition rate and variations in plasma parameters, but a good reproducibility of film thickness and homogeneity was observed over the sampling area.

The p(MA+CNC) nanocomposites with MA/CNC = 1/200 (Figure 1b) formed a nanostructured film having a buckling pattern with an average film thickness of 95 ± 5 nm. According to a screening study of plasma-polymerization parameters, the characteristic morphology of the p(MA+CNC) nanocomposite films was only obtained after deposition at low duty cycles *DC* = 2% as selected in this work, while the higher duty cycles resulted in inhomogeneous mixtures with phase separation between MA and CNCs [21]. The physical and chemical aspects in the formation of the buckling pattern for soft thin films were explained in detail before [51]. The amplitude and wavelength of the buckled structures presently formed on the hydrophilic wafer surfaces are reproducible under the given plasma conditions and are in line with the deposits on the more hydrophobic wafers. It was previously suggested that the CNCs likely act as pinning points for the formation of a metastable film structure and introduce compressive in-plane stresses in the film that cause the buckling effect. As this phenomenon can be observed both on more hydrophobic and hydrophilic wafer substrates, it suggests that the adhesion between the film and the substrate is weak and mainly occurs locally through the CNCs, as hypothesized before [49]. In the previous work, it was indeed suggested to further investigate the role of the interface between the plasma polymer film versus the substrate by varying the substrate chemistry [52], which has been performed in the current study. By comparing both results, it can indeed be concluded that the buckling did not occur through adhesive interface effects, but the buckling rather results from internal stresses in the film introduced by CNCs acting as “defect points” and chemical bonding between the CNCs and MA. 

The pulsed plasma deposits with MA/CNC = 1/500 (Figure 1c) present fiber-like structures without the formation of a coherent film, as indeed the monomer mixture contains abundant CNCs as filler material and a low amount of MA as a film-forming material. The deposition of CNC aggregates is observed after plasma processing, as an activation of the cellulose surfaces likely happens in the plasma reactor while creating reactive species. The interaction between the individual CNCs might result in the formation of large fibrillar entities.

### 2.2. Morphological Analysis of Hydrolyzed Coatings

**Figure 2:**
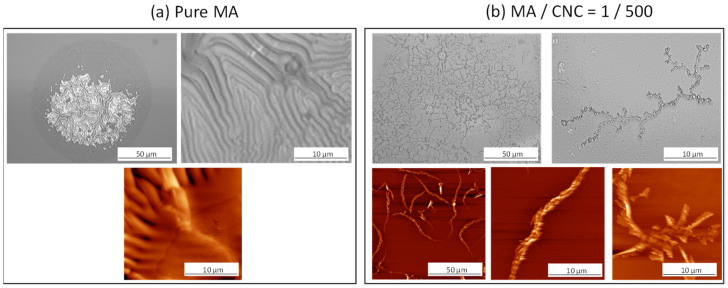
Effect of controlled hydrolysis in controlled moist air (after 4 h) on morphological changes of plasma-polymerized thin films with different composition: (**a**) pure maleic anhydride p(MA) film; (**b**) p(MA+CNC) nanocomposite film with MA/CNC = 1/500, according to optical microscopy (**top row**) and AFM (**bottom row**).

The hydrolysis of p(MA) and p(MA+CNC) films with MA/CNC = 1/500 was performed for times of up to 4 h under a controlled atmosphere of 90% RH and 23 °C; morphological changes are shown in Figure 2, using optical microscopy and AFM. The hydrolysis of pure p(MA) films (Figure 2a) resulted in a break-up into fragments and local swelling, leading to shrinkage. The pure p(MA) films are rather unstable and may show stronger morphological changes after hydrolysis, as reported before [34], owing to the deposition conditions under low *DC* and rather hydrophilic properties of activated silicon substrates, which allow for the migration of moisture within the interface that promotes delamination. In this work, the rearrangement of p(MA) films as formed under low-*DC* pulsed plasma may be favored as the latter conditions imply a relatively low cross-linking density, yielding mobile polymer chains and a fraction of low-molecular species. The nonactivated silicon substrates with more hydrophobic properties, as studied before [49], showed better stability of the p(MA) films. Otherwise, the delamination of p(MA) films on hydrophilic substrates can be expected in parallel with previous studies on the detailed nanoscale effects of water penetration through plasma-polymerized films; the easy migration of water through a hydrophilic film and accumulation of interfacial water allows for easy disruption [35]. Moreover, the low duty cycles *DC* = 2% were selected according to preliminary testing in order to deposit the nanocomposite films with distinct morphology. It is known that p(MA) deposited under low-*DC* conditions are more susceptible to hydrolysis [50]. The instability of the film and shrinkage upon hydrolysis likely originates from the relaxation of internal stresses accumulated during the plasma process [53,54,55] and volume changes [56,57]. The hydrolysis of the films with high CNC content (Figure 2b) also leads to a significant rearrangement of the fibrous structures with the creation of long fibrous networks of entangled fibers. Indeed, the strong interactions between nanocellulose fibers likely occur as a result of the presence of hydroxyl groups at the fiber surface, which create attractive forces in parallel with hydrogen bonding. The difficulties in dispersion and likelihood for agglomeration of nanocellulose fibers is a known phenomenon that corresponds to the observed features after hydrolysis. The good adhesion of the CNC to the substrate was ensured near centers of the cellulose aggregates, as expected through hydrogen bonding [58]. 

**Figure 3:**
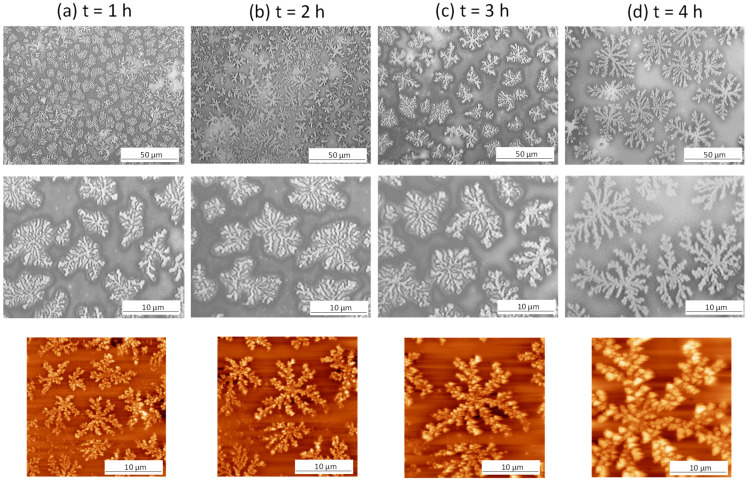
Effect of controlled hydrolysis in controlled moist air on morphological changes of plasma-polymerized thin nanocomposite films p(MA+CNC) with MA/CNC = 1/200 after different times of hydrolysis: (**a**) 1 h, (**b**) 2 h, (**c**) 3 h, (**d**) 4 h, according to optical microscopy (**top rows**) and AFM (**bottom row**).

**Figure 4:**
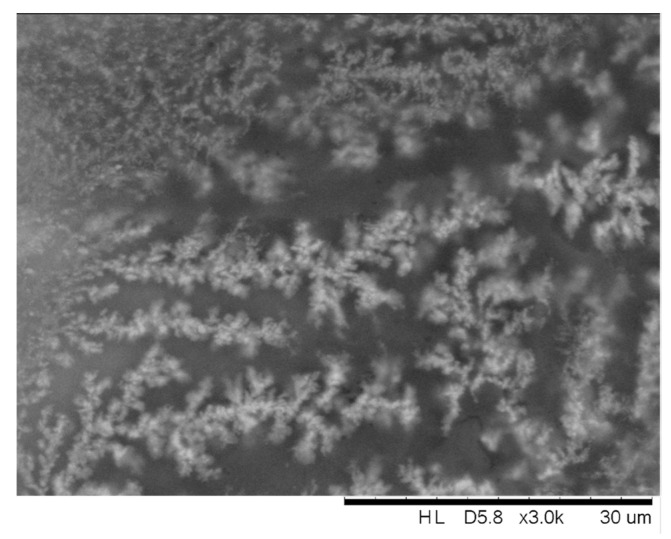
Scanning electron microscopy (SEM) image of crystallized nanocomposite film p(MA+CNC) with MA/CNC = 1/200 after 1 week.

The most interesting features were observed after hydrolysis of nanocomposite films p(MA+CNC) with MA/CNC = 1/200, as illustrated in Figure 3, through optical microscopy and AFM images. A gradual growth of dendritic structures was observed, with the lateral size of the dendrites increasing as a function of hydrolysis time. The similar features remained existing when evaluating the hydrolyzed films by SEM imaging in Figure 4, confirming good stability of the formed crystalline structures after hydrolysis and during analysis under mild vacuum conditions. As the latter measurements were taken after one week of hydrolysis, good stability was ensured while structures were further arranged into larger entities. These unique features are further analyzed in detail during this study. The gradual rearrangement as a function of hydrolysis time was observed, with the formation of growing crystallite structures over time resembling fibrous-like dendrites that may exist of self-assembled branches. The crystalline morphologies are characterized by the branching of the fractals and the local assembly of small crystalline structures that grow into dendritic ring-banded spherulites. The mechanisms for forming such branched dendrites were described before for semicrystalline polymers [59], where tail ends of the lamellae at the growth front induced nucleation sites for the formation of new branches while changing their orientation. The formation of dendritic spherulites with compacted lamellae arranged in multiple arms were typically observed during thermal curing of miscible semicrystalline/amorphous polymer blends that are able to form hydrogen bonds, e.g., with hydroxyl- and carbonyl-containing groups [60]. Alternatively, the current study also illustrates that the presence of foreign nanoparticles introduces similar dendritic morphologies with multiple branching arms radiating from the center. The dendritic crystalline patterns typically result from the diffusion-controlled growth of polymer crystals with the formation of so-called flat-on lamellae in thin films [46]. The crystallization of confined systems in the nanometer-scale range such as thin plasma-polymerized films may generally result in the formation of instable morphologies that are different from traditional spherulite structures observed in bulk materials [61]. The regular spherulites similar to those formed under conditions of bulk crystallization may occur for film thickness larger than 100 nm: these are formed by so-called edge-on lamellae in thicker films [62]. In the present case, the thickness of plasma-deposited nanocomposite films was in the range 30 to 35 nm according to ellipsometry. In thin films, the formation of different crystalline patterns is attributed to the diffusion-controlled processes that govern the crystal growth [63], in contrast to the traditional mechanisms of surface nucleation effects observed in bulk crystallization. The one-dimensional confinement of the growing crystals causes hindrance in the rotational freedom of the lamellae and results in anisotropic crystal growth along preferred orientations [64]. The formation of fractal dendrite (FD) structures as presently observed was reported before as a characteristic crystalline morphology of thin films [65] and was associated with the formation of flat-on lamellar structures. The orientation of flat crystalline lamellae in a confined system was introduced through the combination of the ultrathin thickness of the polymer film and the polymer–substrate interactions. The interactions between the substrate and a thin polymer film were studied in few cases [66]; they seem to have an important effect on the crystalline morphology, as they influence the nucleation and resulting orientation of the crystalline lamellae. In particular, it was found that the widths of the crystalline branches of PCL under thermal crystallization on carbon-coated silica substrates were higher than on bare silica substrates [67]. Also in the present study, the hydrophilic properties of the etched silica wafer substrates enhanced the formation of crystalline dendrites, as the crystallization phenomena were previously not observed for p(MA+CNC) films deposited on nontreated (more hydrophobic) silica wafers [49]. In parallel, it has been demonstrated before that hydrophilic model surfaces enable the diffusion of water at the interface between the substrate and plasma-polymerized films, therefore introducing debonding under wet conditions [52]. The formation of dendrite structures is indeed favored by enhanced mobility of the polymer chains during hydrolysis and arrangement around nucleation sites on the surface. 

The formation of dendritic crystalline structures was not observed after hydrolysis of the p(MA) films due to the presence of CNCs as fillers, which indeed act as nucleating agents that promote local crystallization phenomena [68]. Although the nucleating effect of nanocellulose in confined maleic anhydride films has not yet been demonstrated to the best of our knowledge, the presence of nanocellulose has been already reported to self-assemble in confined droplets [69] and act as a nucleating agent for the formation of dendritic structures in colloidal inorganic systems [70]. The growth of dendrite crystals extends from nucleating sites near the surface of the nanocellulose, while the heterogeneous nucleation conditions and presence of nanocellulose in polymer nanocomposites imply additional confinement of the polymer chains. The heterogeneous microstructures were consequently observed in, e.g., polyethylene oxide/cellulose nanocrystal composites [71]. In particular, the presence of CNCs in plasma-polymerized MA films introduces a number of complex film morphologies that are unstable and difficult to control depending on the plasma-deposition parameters [21], but the progressive growth and complete transformation into stable fractal dendrite structures have not been proven yet. Besides nucleating agents, the CNCs simultaneously act as a stabilizer for the growing crystals, as they function as pinning points to the substrate through the interaction between a high density of hydroxyl groups at the cellulose surface and Si-OH groups at the activated silicon substrates.

### 2.3. Chemical Analysis by XPS

The XPS data for p(MA) and p(MA+CNC) films were analyzed in parallel with known literature for maleic anhydride films [72]. The atomic ratio is calculated from survey XPS wide scans, indicating the presence of oxygen and carbon peaks with complete and homogeneous masking the signals of the silicon substrate, which is expected for a layer thickness above 10 nm (Table 1). 

**Figure 5:**
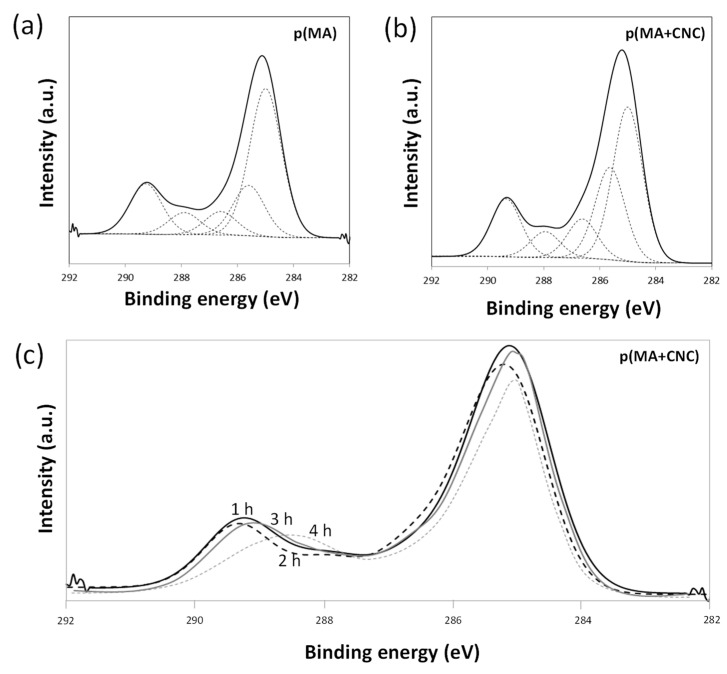
XPS spectra for (**a**) pure p(MA) film before hydrolysis with deconvolution in different components, (**b**) p(MA+CNC) nanocomposite film before hydrolysis with deconvolution in different components, (**c**) p(MA+CNC) nanocomposite films after different hydrolysis times.

The reduction in oxygen concentration indicates the successful formation of a polymerized film during plasma polymerization for both p(MA) and p(MA+CNC), depending on the reaction mechanism and formation of polymerized species under present low-*DC* conditions, as detailed before [17]. The analysis of films before hydrolysis did not show a significant variation in the overall atomic ratio with either changing plasma conditions or introducing CNCs. In particular, the CNCs did not cause a significant amount of unreacted species and/or oxidation of the plasma-polymerized film. The selection of present plasma parameters obviously limited monomer fragmentation and the formation of oxygen-rich species. More detailed analysis of the chemical functionalities through high-resolution XPS spectra of the C1(s) envelope (C-C, C-H hydrocarbon peak of maleic anhydride) is shown for p(MA) in Figure 5a and p(MA+CNC) in Figure 5b, together with values for atomic ratios of different functionalities in Table 2. The envelope C1(s) curve was fitted to five components, with assigned binding energies to each of the hydrocarbon peaks, as accepted in literature [73]. The spectral peaks correspond to various functional groups for which the contributions were calculated from the respective peak areas. The retention of the anhydride moieties was monitored through variations in peak E (C(O)=O), as the highest binding energy represents the presence of anhydride groups [13]. From preliminary testing with different plasma parameters, it was observed that the pulse time affected the intensities of peak E (C(O)=O) that were higher at shorter *t_on_* and longer *t_off_*, leading to an increase in the O/C ratios. The presence of cellulose theoretically does not involve a component in peak E (C(O)=O) due to the absence of carboxylated groups, whose peak heights are therefore used as an internal reference. The esterification in p(MA+CNC) nanocomposite films between cellulose and maleic anhydride is illustrated from XPS analysis by a higher relative intensity of peak B (C-C(O)=O), while the intensity of peak C (C-OH) for nanocomposite films is lower than expected from the superposition of the single spectra of cellulose and maleic anhydride. The intensity ratio of peak C (C-OH) relative to peak D (O-C-O) is less than expected for pure cellulose (5:1) [74], and thus indicates that reactive groups at the cellulose surface reacted during esterification.

The progressive hydrolysis with time causes significant changes in the chemistry of p(MA+CNC), as illustrated by a variation of the XPS spectra in Figure 5c. The XPS spectra indicate a shift in the peak with the highest binding energy as a result of the decreasing intensity of peak E (C(O)=O) and higher contribution of peak D (C=O). The hydrolysis evidently involves the ring-opening reaction of the maleic anhydride ring structure and the formation of C=O moieties that can either be related to free carboxylic or ester moieties. However, the hydrolysis caused no further increase in peak E (C(O)=O) related to formation of free carboxylic groups. Hence, the hydrolysis most likely introduced further esterification between the carboxylic acid groups with the CNC surface. Based on further calculations, about 10% of the hydroxylated maleic anhydride groups remained in the carboxylic acid form, and the rest were supposed to further react with the CNC. This result can be expected, owing to the high reactivity of the hydroxyl groups at the nanocellulose surface. The reactivity can be compared with other reactions and adhesive studies in hydrolyzed plasma-polymerized maleic anhydride films, where a remaining carboxyl content of 8% was reported [75].

### 2.4. Chemical Analysis by FTIR Spectroscopy

**Figure 6:**
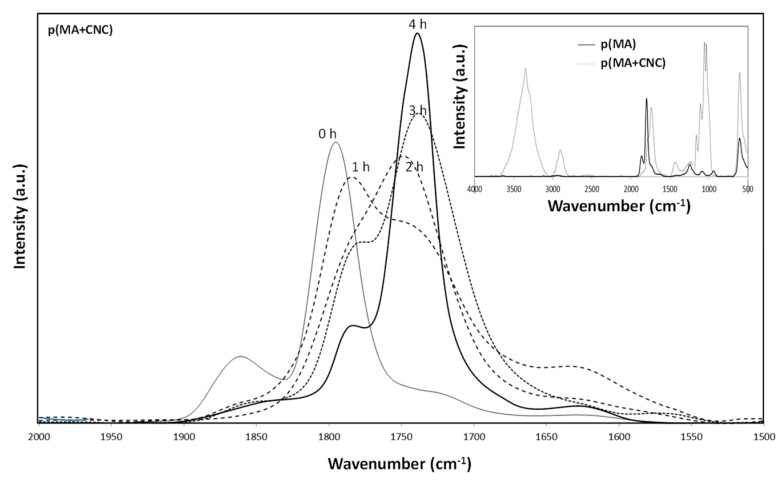
FTIR analysis of p(MA) and p(MA+CNC) films after deposition (inset picture) and effects of progressive hydrolysis of p(MA+CNC) in controlled moist air with times from 0 to 4 h.

The stability of maleic anhydride polymer films can be judged depending on the amount of anhydride groups present, as the films can react with water vapor, resulting in the opening of the anhydride ring and the formation of carboxylic acid groups. The FTIR spectra of p(MA) films and p(MA+CNC) nanocomposite films after deposition (before hydrolysis) are shown as an inset in Figure 6, while the effect of increasing hydrolysis time on the spectra of a p(MA+CNC) film is illustrated in the main figure. The fingerprint area is characterized by anhydride peaks (main peak at 1780 cm^−1^ and shoulder band at 1860 cm^−1^ corresponding to the symmetric and asymmetric carbonyl (C=O) stretch in the anhydride ring), and possibly two additional peaks at 1730 and 1630 cm^−1^ corresponding to the ester and free carbonyl groups [76]. The latter peaks are not observed in the native plasma-polymerized films as a result of appropriate selection of plasma conditions with high retention of the anhydride structures, which agree with the high conversion levels and appropriate selection of plasma conditions in this work. It is indeed confirmed that the intensities of the latter components are low and the anhydride ring remains preserved as the duty cycle decreases [15,17]. Under these conditions, plasma polymerization implies reactivity near the double bond of the MA monomer forming the polymer backbone, in parallel with minimized conversion of the C=O in the five-membered anhydride ring. 

For p(MA), spectral bands are also present at 1240 cm^−1^ (cyclic conjugated anhydride group), 1097 cm^−1^ (C-O-C stretching vibrations) and 964 cm^−1^ (unconjugated anhydride group) [50], which confirm the good retention of anhydride groups under given plasma conditions. For p(MA+CNC), the maleic anhydride cyclic ring structures have partially disappeared, as observed through a shift in characteristic peaks towards 1781 cm^−1^ (ester) and 1735 cm^−1^ (carbonyl) [77]. The latter indicates the esterification between the maleic anhydride and CNC surface during plasma polymerization of the nanocomposite films. The changes in FTIR spectra during hydrolysis with time (1 to 4 h) involve chemical modifications in the nanocomposite films with a shift in spectral bands at wavenumbers related to the ring-closed maleic anhydride structure towards spectral bands at lower wavenumbers related to carboxylic groups. The hydrolysis involves increased intensity of peaks at 1730 and 1630 cm^−1^, corresponding to ester and carbonyl groups. As the latter has not been observed in the p(MA) films, it is unlikely that there is a contribution of unreacted (nonpolymerized) maleic anhydride fragments to this band. A sharp band at 1647 cm^−1^ related to the C=C double bonds of unreacted monomers has not been observed. Indeed, the plasma parameters have been preliminary optimized to obtain the highest monomer conversion.

**Figure 7:**
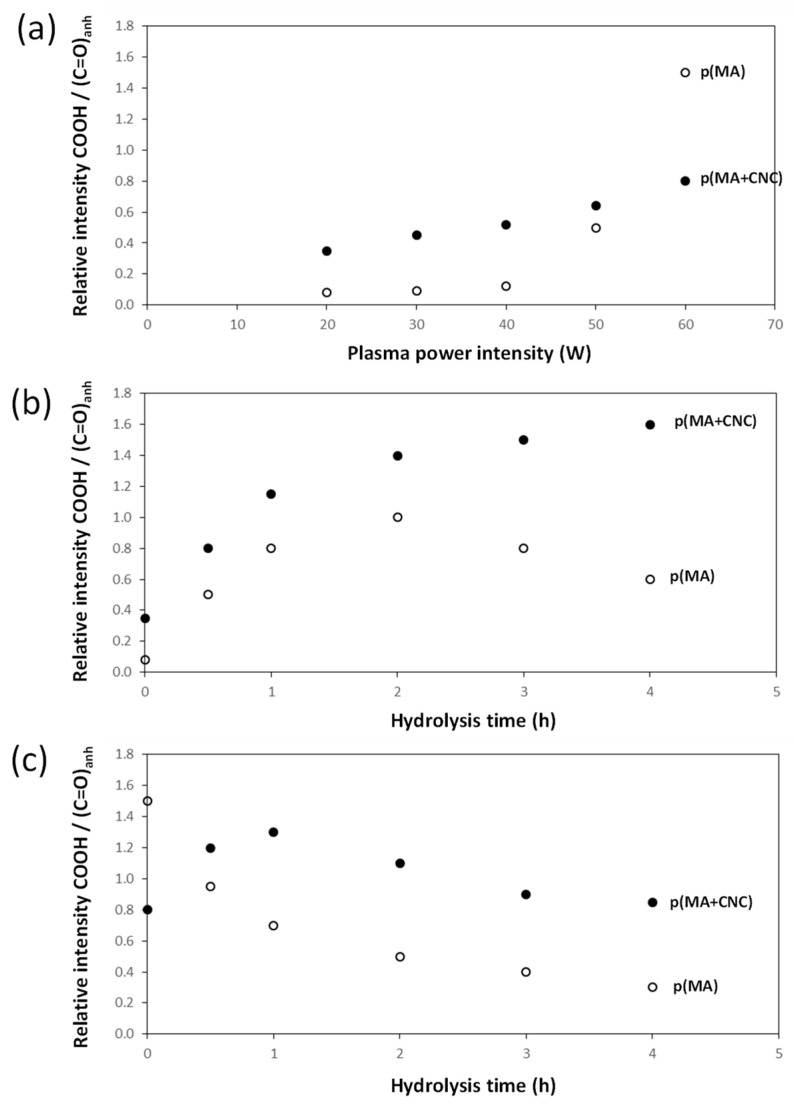
Evolution of chemical structure of the p(MA) films and p(MA+CNC) nanocomposite films based on FTIR analysis: (**a**) effect of plasma conditions (variable power intensity, *DC* = 2%); (**b**) effect of hydrolysis at 20 W, *DC* = 2%; (**c**) effect of hydrolysis at 60 W, *DC* = 2%.

The hydrolysis of p(MA+CNC) and p(MA) films evolves progressively with changes in chemical structure of the film. The loss of the anhydride groups and kinetics are studied below from quantitative analysis of the FTIR spectra through the calculation of relative intensities of specific band ratios, as shown in Figure 7. The chemical changes in the plasma-polymerized films are characterized by considering the band ratio of carbonyl (1630 cm^−1^) and ester (1730 cm^−1^) groups relatively to the anhydride groups (1780 and 1860 cm^−1^), as the formation of ring-opened anhydride moieties implies the formation of carboxylic acid groups. The p(MA) films in humid air were previously found to react with water vapor [17], with kinetics of hydrolysis depending on the selected duty cycle and plasma power. The fast absorption of water is expected in parallel with the hydrophilic properties of the p(MA) films. The kinetics for hydrolysis on present films can be faster compared to other literature results reporting good hydrolysis resistance [13], as low duty cycles have to be applied here for the successful creation of the p(MA+CNC) nanocomposite films. The films created at low duty cycles are generally less cross-linked compared to films deposited at higher duty cycles, leading to the easier penetration of the water molecules, which leads to swelling of the film. 

According to the evolution of hydrolysis kinetics, the following trends are seen:

- The effects of plasma deposition conditions on the chemistry and stability were evaluated (Figure 7a) after deposition of p(MA) and p(MA+CNC) films at different plasma power intensities and *DC* = 2%. In general, the film growth is determined by the formation of a cross-linked polymer network depending on a combination of competitive polymerization and ablation [20]. In particular, the ablation mechanisms become more important and create multiple radical sites, allowing for the formation of a densely cross-linked polymer network at high plasma power. It was also observed for p(MA) films that the formation of ester and carbonyl groups was favored at increased plasma power [33]. The high plasma power consequently results in the creation of films with a more cross-linked polymer network and low penetration rate of water molecules. Otherwise, the structure of the polymer films at low plasma power generally has a more open polymer network with longer polymer chains between the cross-linking points. The differences in the chemistry of deposited films of p(MA) and p(MA+CNC) at plasma power up to 50 W indicate that the p(MA+CNC) films include a higher amount of carboxylic groups owing to the creation of ring-opened anhydride as a result of interface interactions between the maleic anhydride and hydroxyl groups at the surface of CNC. However, the deposition of films with high plasma power of 60 W involves the creation of a higher amount of carboxylic groups relatively to the anhydride carbonyl groups for p(MA), as the relative amount of intact anhydride groups might reduce at a higher plasma power in parallel with the fragmentation of the main polymer chain. Alternatively, the stability of the p(MA+CNC) films is higher at the high plasma power of 60 W, while the anhydride structures in p(MA) films start to fully decompose into ring-opened carboxylic moieties.
- The effects of hydrolysis on p(MA) and p(MA+CNC) films deposited at a low plasma power of 20 W and duty cycle of 2% (Figure 7b) indicate different stability as a function of hydrolysis time. An increase in the concentration of carboxyl groups for the p(MA+CNC) films suggests ongoing ring opening of the anhydride structure during hydrolysis, whereas a decrease in the concentration of carboxylic groups for the p(MA) film after a certain hydrolysis time is attributed to the progressive dissolution of the polymerized film. Therefore, the p(MA) films were less stable to hydrolysis compared to p(MA+CNC) films, as confirmed by previous optical microscopy showing shrinkage of the film over the surface. In general, the application of higher duty cycles is known to reduce the hydrolysis of pure p(MA) films, while the use of low duty cycles implies a lower cross-linking degree and high ability for water penetration during hydrolysis [17]. However, a *DC* = 2% was selected in the present work to form the p(MA+CNC) nanocomposite films with distinct nanostructured morphologies, as described before [64]. A preliminary screening of plasma conditions indeed revealed that the morphology of p(MA+CNC) nanocomposite films was not obtained at high *DC*. The importance of a low *DC* can be understood as the radical species that are formed during the short *t_on_* times, need time for diffusion towards the nucleation sites near CNC during the long *t_off_* times, and initiate the growth of crystalline structures.
- The stability during the hydrolysis of p(MA) and p(MA+CNC) films deposited at high plasma power of 60 W and duty cycle of 2% (Figure 7c) is different to films deposited at low plasma power. The power density indeed has a critical influence on the film structure, where the higher power may lead to polymer films with both higher cross-linking density, and probably forms more hydrolyzable polymer fragments from ring-opened anhydride structures (e.g., ester and carboxyl groups) in the main polymer chain. The hydrolysis of the latter film structures is indeed favored and leads to a progressive decrease in carboxyl groups through dissolution of the film. As before, the stability of the p(MA+CNC) film to dissolution is superior to that of p(MA), as there is initially a regular hydrolysis of the film during the first hour of exposure and the concentration of carboxylic groups remains higher in p(MA+CNC) after longer hydrolysis time.

## 3. Materials and Methods

### 3.1. Materials

**Figure 8:**
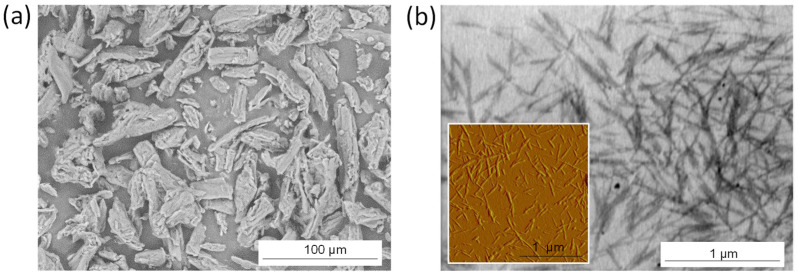
Morphology of cellulose materials used in this research: (**a**) microcrystalline cellulose (MCC) starting material; (**b**) cellulose nanocrystals (CNC) obtained after acid hydrolysis, according to SEM analysis (main pictures) and AFM (inset (**b**)).

The maleic anhydride (MA) was purchased as pellets of high purity (>99.0%) from Sigma Aldrich and ground into a fine powder, followed by overnight drying at 60 °C. The microcrystalline cellulose (MCC, Figure 8a) was purchased from Sigma Aldrich and used as a source for the production of cellulose nanocrystals (CNC, Figure 8b) through hydrolysis with sulfuric acid (63.5%) at 45 °C during 130 min, in agreement with a well-established procedure published before [78]. The CNC was isolated after quenching with ice, subsequent washing with distilled water and a centrifugation step (15,000 rpm, 10 min) in order to remove the excess acid. The final purification was performed in a dialysis operation against distilled water until neutral pH was obtained through removal of excess acid and nonreacted cellulose substances. The purified CNC was suspended in water at a concentration of 1 wt.-% and sonicated for 30 min. The CNC suspension was finally freeze-dried in a benchtop Alpha 1-2 LDPlus lyophilizer (Christ, Osterode, Germany) and characterized with following properties: length = 300 ± 25 nm, diameter 5 ± 1 nm, crystallinity index 80%, degree of substitution 0.05, as calculated from the measured sulfur content.

Three powdery samples were prepared with different weight ratios of MA and CNC, including (i) sample 1: pure MA; (ii) sample 2: MA/CNC = 1/200 (*w*/*w*); and (iii) sample 3: MA/CNC = 1/500 (*w*/*w*). The samples were degassed through repetitive freeze–thaw cycles and stored in vacuum-sealed caps before further use as monomers to be introduced in the plasma reactor. 

Single-side-polished silicon wafers (500 µm thick) were used as substrates for deposition of the plasma-polymerized thin films, which were pretreated and cleaned in piranha solution (mixture of 1/3 (*v*/*v*) of 30% H_2_O_2_ and 98% H_2_SO_4_). The etching provided the removal of contaminants and the creation of an activated surface with hydroxyl groups in order to give good interaction between the substrate and plasma-deposited film. In particular, the native oxidized top layer of SiO_2_ was removed from the silicon wafers and transformed into a hydroxyl-rich surface (SiOH) with hydrophilic properties.

### 3.2. Plasma Coating and Hydrolysis Treatment

The thin films of pure MA or MA/CNC nanocomposites were deposited through pulsed plasma polymerization. A low-power radio-frequency (RF) plasma reactor chamber was used, consisting of a glass cylindrical tube (6 cm diameter × 25 cm length) that is surrounded in the central part by an externally wound copper coil (6 cm diameter × 5 turns) near the position where the substrate is placed. The samples were placed in the center of the reactor vessel on a sample stage. The RF power supply was provided through a high-frequency generator connected to an LC network and matching the output impedance of 13.6 MHz, while the standing wave ratio of the transmitted power was minimized, as visualized by the connected oscilloscope. The plasma reactor was connected with a valve to regulate the gas inlet for monomers at one side. The other side of the vacuum chamber was connected to a Pirani pressure gauge connected with a pump system and liquid nitrogen cold trap. The argon carrier gas was used in a ratio of 90% Ar and 10% monomer, as regulated by the partial pressure of the respective gasses. 

The vacuum chamber was purged with high-power air-plasma treatment (60 W) for cleaning before each experiment, and afterwards operated at a stable pressure of 0.2 mbar with a controlled flow rate of about = 1.6·10^−9^ kg/s, as regulated from variations in monitored pressure variations through a needle valve. The base pressure of the plasma reactor was allowed to stabilize for some minutes before the degassed monomer was introduced in the flow. During introduction of the monomer vapor, sublimation of the mixed monomer powders occurred. The initiated conditions for pulsed-plasma polymerization and corresponding plasma time were optimized from preliminary testing for deposition of the required film morphologies. The peak power of the high-frequency generator was fixed at *P_p_* = 20 or 60 W with a pulse-on time (*t_on_*) of 25 µs, pulse-off time (*t_off_*) of 1200 µs and pulse frequency of 816 Hz, corresponding to a given duty cycle (*DC* = *t_on_*/(*t_on_* + *t_off_*)) of 2% and nominal power delivered to the system *P_nom_* = *P_p_*. *DC* = 0.4 W (20 W) or *P_nom_* = *P_p_*. *DC* = 1.2 W (60 W). As such, the ratio of plasma power to flow rate was selected at *P_nom_*/ = 0.22 W/cm^3^/min (20 W) or *P_nom_*/ = 0.66 W/cm^3^/min (60 W). The power matching was tuned to provide a minimum reflected power as needed for proper control of the pulsing plasma. From preliminary testing, the higher duty cycles (*DC* = 25, 50, 100%) and higher plasma power (80 W) resulted only in single deposits and less homogeneous films. In pulsed-plasma polymerization, the lower duty cycles are indeed preferred, as they promote the retention of chemical functionalities without fragmentation of the monomers [15]. The use of low-duty-cycle conditions for nanostructured films was previously also advised [79], as enough time should be available during *t_off_* for diffusion of the reactive species that are formed during *t_on_*. The film thickness was controlled by fixing the plasma polymerization time at 2500 s and experimentally validated from theoretical calculations based on a deposition rate of 0.012 nm/s. Finally, the RF plasma was switched off at the end of the prescribed plasma polymerization, while the monomer gas flow was continued for another 2 min prior to evacuating the plasma chamber to atmospheric pressure. 

The coated substrates were placed in a humidity-controlled chamber at 23 °C and 90% relative humidity (RH) in order to evaluate stability and influence of hydrolysis on the pulsed-plasma-polymerized films. The hydrolysis times were increased towards 0.5, 1, 2, 3, 4 h, after which the polymer films were further characterized to observe variations in morphology and chemical characteristics.

### 3.3. Sample Characterization

Optical light microscopy was performed on a Metallux II (Leitz, Wetzlar, Germany) at magnifications 20× and 50×. Atomic force microscopy (AFM) was performed on a Nanoscopy IV (Veeco Digital Instruments, Plainview, NY, USA) in tapping mode using a tip with stiffness 48 N/m and resonance frequency of 190 kHz (Nanoworld, type NCLR). The film thickness was measured by ellipsometry (Multiskop M-033k001, Physik Instrumente, Karlsruhe, Germany) using a 532 nm Nd:YAG laser with 1 mm^2^ spot size. The thickness measurements were averaged over five positions distributed over the silicon substrate (10 × 10 mm^2^), and good homogeneity of the film thickness was confirmed. A scanning electron microscope (SEM) image was taken on a tabletop TM3000 (Hitachi, Krefeld, Germany) under medium vacuum pressure (10^−3^ bar).

The high-resolution polarization-modulated infrared reflection–absorption spectroscopy (PM-IRAS) was performed on a IFS 66v/S spectrometer (Bruker Optics GmbH, Karlsruhe, Germany) with liquid-nitrogen-cooled MCT detector, HINDS PEM-90 ZnSe photoelastic modulator and PMA 37 polarization module. The spectra were averaged from 200 scans with a resolution of 4 cm^−1^ in the wavelength region between 4000 to 400 cm^−1^, recording an angle of incidence of 82.5° relative to the surface normal. X-ray photoelectron spectroscopy (XPS) was examined on LHS-11 system (Leybold-Heraeus GmnH, Cologne, Germany) using a Mg Kα line excitation radiation (E = 1253.6 eV) and the C1s energy level (285.0 eV) as a reference. The signal was collected at 90° from the substrate, with electron detection in the constant analyzer energy mode. A survey spectrum was recorded with a pass energy of 500 eV and the high-resolution spectra were recorded at 200 eV. Peak fitting was performed with CASAXPS software, applying mixed Gaussian–Lorentzian (30%) components having equal full-width-at-half-maximum (FWHM). The surface composition (atom-%) was calculated from integrated peak areas of each component and corrected for the transmission factor of the spectrometer, the mean free path and Scofield sensitivity factors of each atom.

## 4. Conclusions

The hydrolysis of pulsed-plasma-deposited nanocomposite films, including maleic anhydride and cellulose nanocrystals (CNC), introduced a unique morphology with progressively growing fractal dendrite crystals over time. CNCs aid in forming the structures because of their nucleating effect and local pinning to the activated substrate. The morphologies were previously not formed on the more hydrophobic silica substrates, illustrating the role of water migration and the interface between the substrate and polymer film introducing delamination. Alternatively, the films of pure maleic anhydride caused shrinkage, and higher concentrations of CNCs introduced agglomeration of the fibers after hydrolysis. In parallel with the selected conditions of pulsed-plasma polymerization at low duty cycles, the deposited films are less stable and form unstable structures that can be converted into more stable crystalline structures after hydrolysis. 

The role of CNCs was further analyzed from further chemical characterization, indicating surface interactions between the maleic anhydride and cellulose after plasma deposition likely through esterification. Furthermore, hydrolysis introduced further esterification between the carboxylic acid groups with the CNC surface, as about 10% of the hydrolyzed maleic anhydride groups remained in the carboxylic acid form and the rest were supposed to further react with the CNCs. The stability of maleic anhydride films against hydrolysis and hydrolysis kinetics were assessed from the conversion of anhydride groups into carboxylic acid groups, where the presence of CNCs improves the retention of anhydride groups in the films after deposition at high plasma power and improves the stability against hydrolysis before dissolution of the film, as more frequently observed for pure maleic anhydride films. 

## Figures and Tables

**Table 1:** XPS elemental composition (at.-%) of plasma-polymerized films (20 W, *DC* = 2%).

Sample	%C	%O	O/C
MA monomer	69.8	30.2	0.43
p(MA) film p(MA+CNC) film	74.8 76.0	25.2 24.0	0.33 0.32

**Table 2:** XPS elemental composition (at.-%) of p(MA) and p(MA+CNC) films based on decomposition of high-resolution C1(s) spectra.

Peak	A	B	C	D	E
**Binding Energy**	**285.0 eV**	**285.7 eV**	**286.6 eV**	**287.9 eV**	**289.3 eV**
	**C-C**	**C-C(O)=O**	**C-O**	**C=O/O-C-O**	**C(O)=O**
p(MA) film p(MA+CNC) film	61.0 41.7	11.0 24.8	11.2 10.6	5.8 7.1	11.0 15.7

## Data Availability

Not applicable.
